# Magnesium increases insulin-dependent glucose uptake in adipocytes

**DOI:** 10.3389/fendo.2022.986616

**Published:** 2022-08-25

**Authors:** Lynette J. Oost, Steef Kurstjens, Chao Ma, Joost G. J. Hoenderop, Cees J. Tack, Jeroen H. F. de Baaij

**Affiliations:** ^1^ Department of Physiology, Radboud Institute for Molecular Life Sciences, Radboud University Medical Center, Nijmegen, Netherlands; ^2^ Laboratory of Clinical Chemistry and Hematology, Jeroen Bosch Hospital, ‘s-Hertogenbosch, Netherlands; ^3^ Beijing Tongren Hospital Beijing Institute of Ophthalmology, Beijing Ophthalmology and Visual Science Key Laboratory, Beijing Tongren Eye Center, Capital Medical University, Beijing, China; ^4^ Department of Internal Medicine, Radboud Institute for Molecular Life Sciences, Radboud University Medical Center, Nijmegen, Netherlands

**Keywords:** Magnesium, type 2 diabetes, insulin resistance, glycemic control, glucose transporter 4

## Abstract

**Background:**

Type 2 diabetes (T2D) is characterized by a decreased insulin sensitivity. Magnesium (Mg^2+^) deficiency is common in people with T2D. However, the molecular consequences of low Mg^2+^ levels on insulin sensitivity and glucose handling have not been determined in adipocytes. The aim of this study is to determine the role of Mg^2+^ in the insulin-dependent glucose uptake.

**Methods:**

First, the association of low plasma Mg^2+^ with markers of insulin resistance was assessed in a cohort of 395 people with T2D. Secondly, the molecular role of Mg^2+^ in insulin-dependent glucose uptake was studied by incubating 3T3-L1 adipocytes with 0 or 1 mmol/L Mg^2+^ for 24 hours followed by insulin stimulation. Radioactive-glucose labelling, enzymatic assays, immunocytochemistry and live microscopy imaging were used to analyze the insulin receptor phosphoinositide 3-kinases/Akt pathway. Energy metabolism was assessed by the Seahorse Extracellular Flux Analyzer.

**Results:**

In people with T2D, plasma Mg^2+^ concentration was inversely associated with markers of insulin resistance; i.e., the lower Mg^2+^, the more insulin resistant. In Mg^2+^-deficient adipocytes, insulin-dependent glucose uptake was decreased by approximately 50% compared to control Mg^2+^condition. Insulin receptor phosphorylation Tyr1150/1151 and PIP3 mass were not decreased in Mg^2+^-deficient adipocytes. Live imaging microscopy of adipocytes transduced with an Akt sensor (FoxO1-Clover) demonstrated that FoxO1 translocation from the nucleus to the cytosol was reduced, indicting less Akt activation in Mg^2+^-deficient adipocytes. Immunocytochemistry using a Lectin membrane marker and at the membrane located Myc epitope-tagged glucose transporter 4 (GLUT4) demonstrated that GLUT4 translocation was diminished in insulin-stimulated Mg^2+^-deficient adipocytes compared to control conditions. Energy metabolism in Mg^2+^ deficient adipocytes was characterized by decreased glycolysis, upon insulin stimulation.

**Conclusions:**

Mg^2+^ increases insulin-dependent glucose uptake in adipocytes and suggests that Mg^2+^ deficiency may contribute to insulin resistance in people with T2D.

## Introduction

T2D is a chronic condition that is characterized by insulin resistance and relative insulin deficiency (1), leading to microvascular and macrovascular complications (2–4). Insulin resistance enhances the hepatic glucose production and diminishes glucose uptake in insulin-sensitive cells, including adipose tissue, skeletal muscle and liver tissue (5). Reduced insulin action can be compensated by increased insulin secretion, but when insulin production capacity is jeopardized, chronic hyperglycemia will develop (6).

In healthy people, high glucose levels induce the secretion of insulin. Upon insulin binding autophosphorylation of the insulin receptor (IR) occurs and the phosphoinositide 3-kinases/Akt (PI3K/Akt) pathway is activated resulting in GLUT4-mediated glucose uptake in adipocytes and skeletal muscle cells (7–9). The insulin receptor substrates (IRS) active the PI3K enzyme, which converts phosphatidylinositol-2,4,5-triphosphate (PIP2) to phosphatidylinositol-3,4,5-triphosphate (PIP3) (10). Subsequently, PIP3 interacts with Akt to phosphorylate Akt substrate of 160 kDa (AS160) resulting in translocation of GLUT4 to the cell membrane (11). Insulin resistance decreases glucose uptake that may be attributed, at least partially, to defects in PI3K/Akt signaling and GLUT4 translocation (9, 12, 13).

The prevalence of hypomagnesemia (plasma/serum Mg^2+^< 0.7 mmol/L) in T2D is between 9.1–47.7%, which is tenfold higher compared to the healthy population (14–21). Hypomagnesemia may contribute to insulin resistance, as Mg^2+^ supplementation enhances insulin sensitivity and glucose profiles both in people with and without diabetes (22–25). Supplementation of Mg^2+^ increases IR and GLUT4 levels in rat skeletal muscle (26, 27). However, the mechanism of Mg^2+^ in the insulin-dependent glucose uptake has not been studied in adipocytes.

In this study we aimed to unravel the role of Mg^2+^ deficiency in insulin-dependent glucose uptake. We first studied the association of plasma Mg^2+^ with insulin resistance markers in people with T2D. Next, we assessed the molecular mechanism of Mg^2+^ on IR phosphorylation, PIP3 mass generation, Akt activation, GLUT4 translocation and energy homeostasis in mature 3T3-L1 adipocytes.

## Materials and methods

### Type 2 diabetes cohort statistics

Details about the Diabetes Pearl Cohort have previously been reported (18). In short, 395 people with T2D were included and plasma samples were taken after an overnight fasting period and immediately analyzed for laboratory parameters (glycated hemoglobin (HbA_1c_), plasma glucose, creatinine, total cholesterol, triglycerides (Ty), high-density lipoprotein (HDL), low-density lipoprotein (LDL) and magnesium. Body mass index (BMI) was calculated as kilograms per body mass. Waist circumference was measured after normal exhalation in duplicate and repeated if the difference was >1.0 cm. For this study we used the Triglyceride Glucose-Body Mass Index (TyG-BMI), Triglyceride Glucose-Waist Circumference (TyG-WC) and Triglyceride HDL (Ty/HDL) ratio as surrogate markers for insulin resistance by using the following calculations: TyG-BMI=Ln [Ty (mg/dL) * plasma glucose (mg/dL)/2] * BMI, TyG-WC= Ln [Ty (mg/dL) * plasma glucose (mg/dL)/2] × WC (cm) and Ty/HDL ratio= Ty (mg/dL)/HDL (mg/dL) (28).

### Cell culture

The 3T3-L1 fibroblasts (ATCC, CL-173) were cultured in Dulbecco’s modified Eagle’s medium (DMEM) (Lonza Westburg, Leusden, the Netherlands) supplemented with 4 mmol/L L-glutamine (GE healthcare Life Sciences, Logan, UT, USA), MEM Non-Essential Amino Acids (Lonza Westburg) and 10% (v/v) fetal bovine serum (FBS) (Greiner Bio One, Alphen aan den Rijn, the Netherlands), at 37°C in a humidified atmosphere of 5% (v/v) CO_2_ in air. Cells were induced two days post-confluence by adding 1 µg/ml bovine insulin (Sigma-Aldrich, St. Louis, MO, USA), 0.5 mmol/L 3-isobutyl-1-methylxanthine (IBMX) (Sigma-Aldrich), and 1 µM dexamethasone (Sigma-Aldrich) for 3 days. Cells were incubated for 4 days with cultured medium supplemented with 1 µg/ml bovine insulin only (refreshed after 2 days) to obtain fully differentiated adipocytes. Adipocytes were maintained another 48 hours in culture medium followed by a 24 hour incubation with either 1 or 0 mmol/L Mg^2+^ (Sigma-Aldrich) DMEM medium (containing 25 mmol/L D-glucose, 0.04 mmol/L Phenol Red, 1 mmol/L sodium pyruvate, 1.8 mmol/L CaCl2, 0.00025 mmol/L Fe(NO3)3, 17.9 mmol/L NaHCO3, 5.3 mmol/L KCl, 110.3 mmol/L NaCl, 0.9 mmol/L NaH2PO4, 0.4 mmol/L glycine, 0.4 mmol/L L-Arginine hydrochloride, 0.2 mmol/L L-Cystine 2HCl, 4 mmol/L L-Glutamine, 0.2 mmol/L L-Histidine hydrochloride, 0.8 mmol/L L-Isoleucine, 0.8 mmol/L L-Leucine, 0.8 mmol/L L-Lysine hydrochloride, 0.2 mmol/L L-Methionine, 0.4 mmol/L L-Phenylalanine, 0.4 mmol/L L-Serine, 0.8 mmol/L L-Threonine, 0.08 mmol/L L-Tryptophan, 0.4 mmol/L L-Tyrosine disodium salt dihydrate, 0.8 mmol/L L-Valine, MEM Vitamin Solution (Thermo Fisher Scientific, Waltham, MA USA) and 10% (v/v) FBS). Cells were cultured without FBS after 18 hours of incubation, 6 hours prior to the experiment.

To transduce 3T3-L1 adipocytes with FoxO1-Clover and Myc-GLUT4-mCherry DNA, lentiviruses were generated using HEK293-FT cells. HEK293-FT cells (kind gift from Translational Metabolic Laboratory, Radboud University Medical Center, bought at Thermo Fisher Scientific, R70007) were grown in DMEM (Lonza Westburg) supplemented with 6 mmol/L L-glutamine (GE healthcare Life Sciences), MEM Amino Acids (Lonza Westburg), 10% (v/v) FBS (Greiner Bio One), 1 mmol/L sodium pyruvate and 1% (v/v) penicillin/streptomycin (10,000 units/10 mg). Cells were maintained at 37°C in a humidified atmosphere of 5% (v/v) CO_2_ in air.

### Radioactive _3_H-2-deoxyglucose uptake

The 3T3-L1 fibroblasts were seeded on coated Poly-L-lysine (PLL) (Sigma-Aldrich) dishes and differentiated. Mature 3T3-L1 adipocytes were washed twice with warm Krebs–Ringer–phosphate–HEPES (KRPH) buffer (20 mmol/L HEPES, 5 mmol/L KH_2_PO_4_, 1 mmol/L CaCl_2_, 136 mmol/L NaCl, and 4.7 mmol/L KCl set at pH 7.4 with NaOH) containing either 1 or 0 mmol/L Mg^2+^. Cells were incubated without or with 10 nmol/L human insulin (Sigma-Aldrich) and 0 or 1 mmol/L Mg^2+^ for 0, 10, 20 or 30 minutes at 37°C in radioactive buffer containing 5 mmol/L 2-deoxyglucose (2DG) (Sigma-Aldrich) and 1 μCi ^3^H-2DG (PerkinElmer, Hoogvliet Rotterdam, the Netherlands). The incubation was stopped by washing with cold KRPH buffer and cells were lysed with 0.05% (w/v) SDS (MP Biomedicals) in dH_2_O. Cell lysates were added to Opti-Fluor O scintillation liquid (PerkinElmer) and counted using the Hidex 600 SL. Radioactive counts were corrected for the background count in each of the incubation buffers. The assay was repeated without the addition of ^3^H-2DG to correct for protein concentrations using the bicinchoninic acid (BCA) assay (Thermo Fisher Scientific) according to the manufacturer’s protocol.

### ELISA assays

3T3-L1 fibroblasts were seeded and differentiated in PLL coated 10 cm dishes or T-175 flasks. Mature adipocytes were stimulated with or without 10 nmol/L human insulin (Sigma-Aldrich) 30 minutes at 37°C. One confluent 10 cm dish per experimental condition was used for the Phospho-Insulin Receptor b (Tyr1150/1151) Sandwich ELISA (Cell Signaling Technology, Leiden, the Netherlands) and one confluent T-175 flask per experimental condition was used for the PIP3 Mass Elisa (Echelon Biosciences, Salt Lake City, UT, USA) were used according to the manufacturer’s protocol.

### Production of recombinant lentivirus

The pLenti-FoxO1-Clover (Addgene, Cambridge, MA, USA) and pLenti-Myc-GLUT4-mCherry (Addgene, Cambridge, MA, USA) plasmids were used as template. The packaging plasmids pLP1, pLP2 and pLP/VSVG were a kind gift from the Translational Metabolic Laboratory, Radboud University Medical Center. HEK293-FT cells were seeded at 70% confluence in Petri dishes coated with 20 μg/ml collagen (Thermo Fisher Scientific) per cm^2^. After cell attachment medium was switched to advanced DMEM (Thermo Fisher Scientific) supplemented with 6 mmol/L L-glutamine (GE healthcare Life Sciences), 2% (v/v) FBS (Greiner Bio One) and 10 μmol/L water-soluble cholesterol (Sigma-Aldrich). After 24 hours of incubation with cholesterol containing medium, HEK293-FT cells were transduced by incubating overnight in Opti-MEM I (Thermo Fisher Scientific) containing 3 μg lentiviral vector and packaging plasmids combined with a 1:3 DNA-reagent ratio Lipofectamine 2000 (Thermo Fisher Scientific) per 100 mm plate. The following morning medium was switched to cholesterol containing medium and incubated 48 hours at 37°C in a humidified atmosphere of 5% (v/v) CO_2_ in air. Lentivirus was harvested by collecting the medium supernatant, followed by centrifugation 5 minutes, 2000 xg at 4°C. The supernatant was filtered using sterile low protein binding 0.45 μm filters (Sigma-Aldrich) and concentrated using Lenti-X Concentrator (Takara Bio, Saint-Germain-en-Laye, France), according to the manufacturer’s protocol. Viral titer was determined using Lenti-X GoStix Plus (Takara Bio), according to the manufacturer’s protocol.

### Lentivirus transduction

Viral supernatants containing FoxO1-Clover or Myc-GLUT4-mCherry were added to fresh medium supplemented with 8 μg/ml Polybrene (Sigma-Aldrich). Mature 3T3-L1 adipocytes were incubated with approximately 40 ng p24 particles per 96 well or 120 ng p24 particles per 24 well and incubated overnight. The next day, the medium was replaced with fresh medium. Microscopy experiments were performed 4 to 6 days post transduction.

### Akt sensor

3T3-L1 fibroblasts were differentiated on fibronectin (Roche Applied Science, Almere, the Netherlands) coated 96 well plates and differentiated. Mature adipocytes were transduced with Lentivirus particles containing FoxO1-Clover. FoxO1 is a direct and specific target of Akt; FoxO1 rapidly translocate from the nucleus to the cytoplasm in response to Akt activation (29). The transduction was followed by a 24 hour incubation with 0 or 1 mmol/L Mg^2+^ of which the last 6 hours adipocytes where incubated without FBS. Medium was replaced 30 minutes prior to starting live imaging; containing 0 or 1 mmol/L Mg^2+^, FBS free, phenol-free, supplemented with 1 μg/ml Hoechst 33342 (Sigma-Aldrich). Live imaging was performed using a ZEISS Axio Observer light microscope. Adipocytes were imaged for 30 minutes with a photo interval per 30 seconds. The cells were stimulated using a final concentration of 10 nM human insulin (Sigma-Aldrich) which was added between 30-60 seconds after the start of visualization. The relative intensity of fluorescent units in the cytosol/nucleus was analyzed by the following formula:


$$\frac{\left((\text{cytosol flourescence}-\text{background flourescence})*\text{area cytosol})-((\text{nuclues flourescence}-\text{background flourescence})*\text{area nucleus}\right)}{\left((\text{nucleus flourescence}-\text{background flourescence})*\text{area nucleus}\right)}$$

All values are normalized by dividing by the first measurement (t=0). Data was fitted using the four-parameter sigmoid dose-response curve. The maximum top value of this function was used as the fluorescent unit’s cytosol/nucleus ratio.

### Immunocytochemistry of endogenously expressed GLUT4

3T3-L1 fibroblasts were seeded on fibronectin (Sigma-Aldrich) coated glass coverslips in 6 well plates. After 24 hours of 0 or 1 mmol/L Mg^2+^ incubation, adipocytes were stimulated with or without 10 nM human insulin (Sigma-Aldrich) 30 minutes at 37°C and subsequently cooled on ice. Subsequently, adipocytes were rinsed in PBS and incubated 10 minutes with 5 ug/ml Lectin Alexa 680 (Thermo Fisher Scientific) in DMEM supplemented with 30 mmol/L HEPES (Sigma-Aldrich), rinsed with PBS and fixated with 4% (w/v) PFA methanol-free formaldehyde solution (Thermo Fisher Scientific). The fixation solution was removed by rinsing with PBS. Adipocytes were permeabilized in 0.3% (v/v) Triton X-100 (Sigma-Aldrich) with 0.1% (w/v) BSA (Sigma-Aldrich) in PBS for 10 minutes. Quenching was done by 50 mmol/L NH_4_Cl in PBS for 10 minutes and followed by washing with PBS. Adipocytes were incubated in 16% (v/v) goat serum (Vector Laboratories, Amsterdam, the Netherlands) with 0.3% (v/v) Triton X-100 (Sigma-Aldrich) for 30 minutes and, subsequently, incubated with primary 1:200 GLUT4 (Santa Cruz Biotechnology, Heidelberg Germany) overnight at 4°C. The following day, cells were rinsed with PBS and incubated with 1:300 secondary Goat anti-Mouse IgG Alexa 488 (Thermo Fisher Scientific) in 16% (v/v) goat serum with 0.3% (v/v) Triton X-100 for 1 hour at room temperature, followed by PBS washing. Nuclei were stained using 0.1 ug/ml DAPI (Thermo Fisher Scientific) for 10 minutes at room temperature, followed by PBS washing and mounting using Fluoromount-G (SouthernBiotech, Birmingham, AL, USA). Fluorescence confocal microscopy was performed with a Zeiss LSM880 and images were taken with the Zeiss Zen software. Co-localization of GLUT4 and Lectin was quantified using ImageJ (JACop) software with the Mander's coefficient.

### Immunocytochemistry of overexpressed GLUT4

Differentiated 3T3-L1 were seeded on fibronectin (Roche Applied Science) coated glass coverslips in 24 well plates. Adipocytes were transduced with Lentivirus particles containing Myc-GLUT4-mCherry. After 24 hours of 0 or 1 mmol/L Mg^2+^ incubation, adipocytes were stimulated with or without 10 nmol/L human insulin (Sigma-Aldrich) 30 minutes at 37°C and subsequently cooled on ice. The adipocytes were fixated with 4% (w/v) PFA methanol-free formaldehyde solution (Thermo Fisher Scientific). Quenching was done by 50 mmol/L NH_4_Cl in PBS for 10 minutes, followed by washing with PBS. Adipocytes were incubated in 16% (v/v) goat serum (Vector Laboratories) with 0.3% (v/v) Triton X-100 (Sigma-Aldrich) for 30 minutes and, subsequently incubated with 1:200 c-Myc Antibody (Santa Cruz Biotechnology). The following day adipocytes were incubated with 1:500 secondary antibody Goat anti-Mouse IgG Alexa 488 (Thermo Fisher Scientific) in 16% (v/v) goat serum with 0.3% (v/v) Triton X-100 for 1 hour at room temperature. Nuclei were stained using 0.1 ug/ml DAPI (Thermo Fisher Scientific) for 10 minutes at room temperature, followed by PBS washing and mounting using Flouromount-G (SouthernBiotech). Fluorescence confocal microscopy was performed with a Zeiss LSM880 and images were taken with the Zeiss Zen software. Co-localization of Myc and GLUT4-mCherry was quantified using ImageJ (JACop) software with the Mander's coefficient.

### Seahorse XF Glycolytic Stress Test

3T3-L1 fibroblasts were differentiated in fibronectin coated XF96 Cell Culture Seahorse plates (Agilent Technologies, Amstelveen, the Netherlands). The sensor cartridge was hydrated 24 hours in XF Calibrant at 37°C in a non-CO_2_ incubator overnight. The culture medium was replaced 1 hour before the Seahorse run with 0 or 1 mmol/L Mg^2+^ DMEM (recipe described in section **Cell Culture**), containing no glucose or pyruvate sources and 5 mmol/L HEPES. The XF96 cell plate was incubated at 37°C in a non-CO_2_ environment for 1 hour before starting the glycolytic stress test. During the run, cells were sequentially treated with or without 10 nmol/L human insulin (Sigma-Aldrich), 10 mmol/L D-glucose, 1 μM oligomycin (Sigma-Aldrich) and 50 mmol/L 2DG (Sigma-Aldrich). Protein concentration was measured using the U/CSF protein kit (Thermo Fischer Scientific). Adhered cells were stored at -80°C until measurement in 0.33% (v/v) Triton X-100 dissolved in 10 mmol/L Tris-HCl (pH 7.6). On the day of measurement, U/CSF protein quantification solution was added to the well. After 10 minutes of incubation at 37°C, absorbance was measured at 600 nm. Protein concentrations in wells containing samples were obtained using the concentration calculated from the sCal (Thermo Fischer Scientific) calibration curve included in each plate. All data was normalized for the amount of protein in each well. The non-glycolytic acidification rate was calculated by: the average of the last rate measurement prior to insulin injection. Glycolysis was calculated by: (maximum rate measurement before oligomycin injection) – (last rate measurement before glucose injection). The glycolytic capacity was calculated by: (maximum rate measurement after oligomycin injection) – (last rate measurement before glucose injection).

### Statistical analyses

All *in vitro* data are presented as mean ± SEM. Statistical significance was evaluated using Two‐Way Analysis of Variance (ANOVA) or by a Student’s T-Test when comparing only two groups. Pearson correlations were performed using SPSS for Windows (V25.0.0.1 IBM) to determine the association between plasma Mg^2+^ concentration and fasting glucose and the insulin resistance markers: TyG-BMI, TyG-WC and Ty/HDL. Normal distribution of data was verified by calculating the quotient of the skewness divided by its standard error. Variables that were not normally distributed were log transformed. Statistical significance set at *P<0.05; **P<0.01; and ***P<0.001.

## Results

### The plasma magnesium concentration is inversely associated with insulin resistance

**Figure 1:**
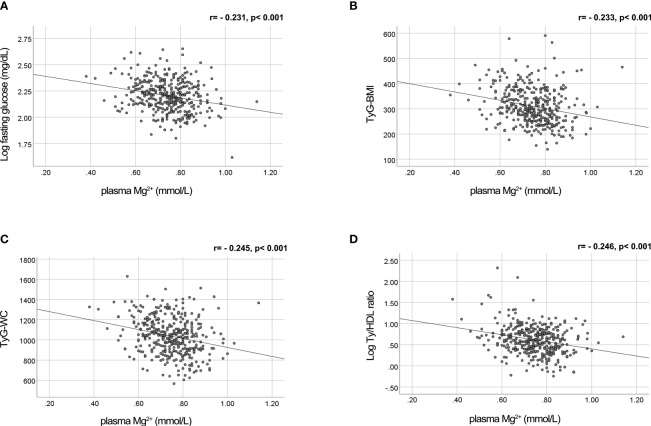
Mg^2+^ is negatively correlated with fasting glucose levels and insulin resistance markers TyG-WC, TyG-BMI and Ty/HDL ratio in people with T2D. Plasma Mg^2+^ concentration scatter plotted versus **(A)** Log fasting glucose (n=382); **(B)** TyG-BMI (n=379); **(C)** TyG-WC (n=376); and **(D)** Log Ty/HDL ratio (n=384) in people with T2D. Statistically analyzed using Pearson correlation. Mg^2+^, magnesium; T2D, type 2 diabetes; TyG-BMI, Triglyceride Glucose-Body Mass Index; TyG-WC, Triglyceride Glucose-Waist Circumference; Ty/HDL, Triglyceride High Density Lipoprotein.

Previously, we demonstrated that plasma triglycerides and fasting glucose levels are major determinants of the plasma Mg^2+^ concentration (18). The negative association of plasma Mg^2+^ concentration with fasting glucose levels is visualized in **Figure 1A**. **Figures 1B–D** shows the negative association of plasma Mg^2+^ and markers of insulin resistance, confirming the association between low Mg^2+^ levels and insulin resistance in people with T2D.

### Magnesium increases insulin-dependent glucose uptake

**Figure 2:**
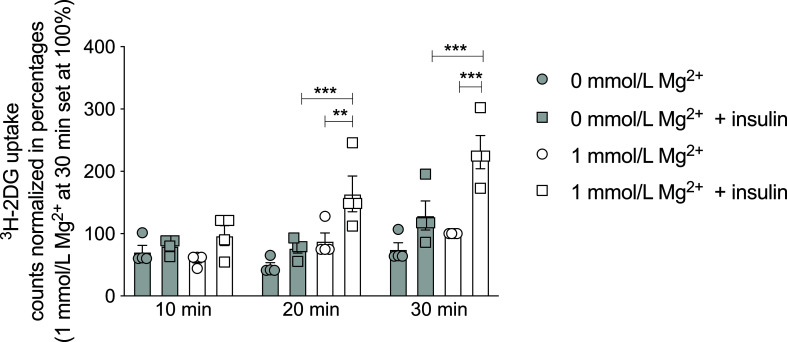
Effect of Mg^2+^ on insulin-dependent glucose uptake in 3T3-L1 adipocytes. ^3^H-2DG uptake for 10, 20 or 30 minutes in 3T3-L1 adipocytes that were incubated with 0 or 1 mmol/L Mg^2+^ for 24 hours (n=4). Legend: circle is without insulin, square is with insulin, grey color is 0 mmol/L Mg^2+^ and white color is 1 mmol/L Mg^2+^. Data are shown as mean ± S.E.M. Statistically analyzed with Two-Way ANOVA. Significance **p<0.01; ***p< 0.001. 2DG = 2-deoxyglucose, Mg^2+^, magnesium; min, minutes.

To determine the potential causative relationship between Mg^2+^ and insulin resistance, the effects of Mg^2+^ on insulin sensitivity and glucose handling were further examined *in vitro*. Radioactive ^3^H-2DG uptake was measured in mature 3T3-L1 adipocytes that were Mg^2+^-deficient (0 mmol/L Mg^2+^) or incubated with its physiological concentration (1 mmol/L Mg^2+^). In Mg^2+^-deficient adipocytes, insulin-stimulated glucose uptake was decreased by 50% at 20 and 30 minutes compared to adipocytes with a physiological Mg^2+^ concentration (P< 0.001) (**Figure 2**).

### Magnesium increases translocation of endogenous and overexpressed GLUT4 upon insulin-stimulation

**Figure 3:**
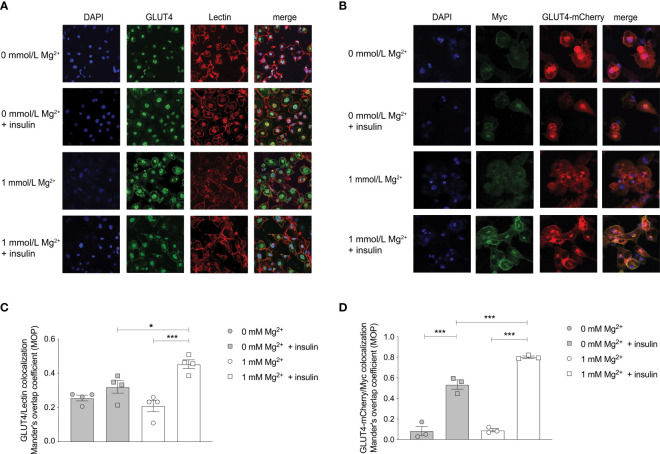
Effect of Mg^2+^ on insulin-dependent localization of the GLUT4 transporter to the cell membrane in 3T3-L1 adipocytes. **(A)** Immunocytochemistry staining of DAPI, GLUT4 and the cell membrane marker Lectin in insulin-stimulated 3T3-L1 adipocytes that are incubated with 0 or 1 mmol/L Mg^2+^ (n=4). GLUT4 signal was assessed at the plasma membrane, since GLUT4 antibody did result in non-specific nuclear staining. **(B)** Immunocytochemistry staining of DAPI, anti-myc and GLUT4-mCherry in insulin-stimulated 3T3-L1 adipocytes that were transduced with pLenti-Myc-GLUT4-mCherry, incubated 0 or 1 mmol/L Mg^2+^ (n=3). **(C)** Quantification of the immunocytochemistry staining by co-localization GLUT4 and Lectin at the plasma membrane compared to total cytosol expression (n=4). **(D)** Quantification of the ratio of cell surface Myc signal to total mCherry signal in 3T3-L1 adipocytes transduced with pLenti-myc-GLUT4-mCherry (n=3). Legend: circle is without insulin, square is with insulin, grey color is 0 mmol/L Mg^2+^ and white color is 1 mmol/L Mg^2+^. Data are shown as mean ± S.E.M. Statistically analyzed with Two-Way ANOVA. Significance *p<0.05; ***p< 0.001. GLUT4, glucose transporter 4; Mg^2+^, magnesium.

Insulin-dependent glucose uptake is mainly mediated by GLUT4 in adipocytes (9, 30). GLUT4 expression at the plasma membrane was increased in insulin-stimulated adipocytes with physiological Mg^2+^ compared to insulin-stimulated Mg^2+^-deficient adipocytes (**Figures 3A, B**). The GLUT4 co-localization with membrane marker Lectin was increased twofold in insulin-stimulated adipocytes incubated with physiological Mg^2+^ compared to Mg^2+^-deficient adipocytes (P< 0.05). The effect of insulin on the translocation of endogenous GLUT4 was almost completely abolished in Mg^2+^-deficient adipocytes (**Figure 3C**). In adipocytes that overexpressed Myc-GLUT4-mCherry and were stimulated with insulin, there was less GLUT4 translocation to the plasma membrane in Mg^2+^-deficient adipocytes compared to controls (P< 0.001), but was not completely abolished (**Figure 3D**). The GLUT4 translocation to the plasma membrane was increased by threefold in insulin-stimulated Mg^2+^-deficient adipocytes and increased by fourfold in insulin-stimulated controls, compared to non-insulin stimulated adipocytes (**Figure 3D**).

### Magnesium is not involved in the autophosphorylation of the insulin receptor

**Figure 4:**
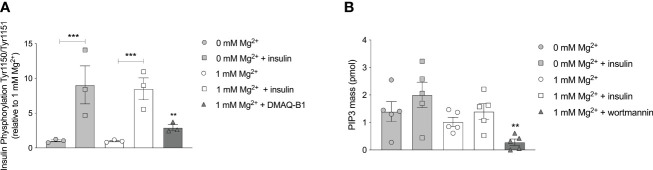
Effect of Mg^2+^ on the auto phosphorylation of the IR at residue Tyr1150/1151 and PI3K kinase regulatory subunits that phosphorylate PIP2 to PIP3. **(A)** The phosphorylation of Tyr 1150/115 in 3T3-L1 adipocytes incubated with 0 or 1 mmol/L Mg^2+^. DMAQ-B1 is used as positive control to mimic IR phosphorylation (n=3). **(B)** PIP3 mass in 3T3-L1 adipocytes incubated with 1 or 0 mmol/L Mg^2+^. Wortmannin is used as positive control to inhibit PI3K enzyme activity (n=5). Legend: circle is without insulin, square is with insulin, grey color is 0 mmol/L Mg^2+^, white color is 1 mmol/L Mg^2+^ and rectangles are positive controls. Data are shown as mean ± S.E.M. Statistically analyzed with Two-Way ANOVA. Positive controls are analyzed with Student t-test, compared to 1 mmol/L Mg^2+^ condition. **p<0.01; ***p< 0.001. Mg^2+^, magnesium; PIP3, phosphatidylinositol-3,4,5-trisphosphate; DMAQ-B1, demethylasterriquinone B1.

Phosphorylation of IR tyrosine residues is the first essential requirement for insulin signaling, with phosphorylation of Tyr1150/Tyr1151 predominant for activation *in vivo* (31, 32). Insulin increased Tyr1150/1151 autophosphorylation in adipocytes (P< 0.001), though this increase was similar in adipocytes with a physiological Mg^2+^ concentration compared to Mg^2+^-deficient adipocytes (**Figure 4A**). Mg^2+^ *per se* had no effect on Tyr1150/1151 autophosphorylation, with and without insulin stimulation. The positive control, DMAQ-B1, a selective stimulator of IR tyrosine kinase phosphorylation, resulted in specific response by increasing Tyr1150/1151 phosphorylation almost threefold compared to the physiological condition (1 mmol/L Mg^2+^ without insulin stimulation) (P< 0.01) (33).

### PIP3 generation is not increased by magnesium

Cell lipid extractions for enzymatic assays were performed to measure PIP3 mass of 3T3-L1 adipocytes incubated with a physiological Mg^2+^ concentration versus Mg^2+^-deficient. Mg^2+^ did not increase PIP3 mass generation. PIP3 mass was not increased in insulin-stimulated adipocyte conditions, with and without Mg^2+^. The positive control, the PI3K enzyme inhibitor wortmannin, decreased PIP3 mass by approximately 80% compared to the physiological condition (1 mmol/L Mg^2+^ without insulin stimulation) (P< 0.05) (**Figure 4B**).

### Magnesium is essential for insulin-stimulated Akt activation

**Figure 5:**
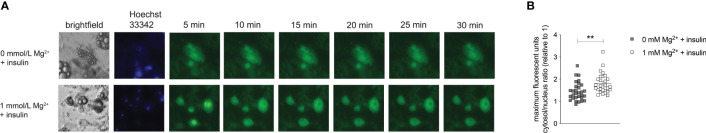
Effect of Mg^2+^ on insulin-dependent Akt activation. Live imaging of insulin-stimulated 3T3-L1 adipocytes transduced with pLenti-FoxO1-Clover and incubated with 0 or 1 mmol/L Mg^2+^. **(A)** Live images showing Brightfield, Hoechst 33342 and Clover signal in 3T3T-L1 adipocytes stimulated with insulin. **(B)** Quantified maximal mean intensity ratio of the cytosol in 3T3-L1 adipocytes stimulated with insulin (n=5, 3–10 cells analyzed per biological replicate). Statistically analyzed with Student’s T-Test. grey color is 0 mmol/L Mg^2+^ and white color is 1 mmol/L Mg^2+^. Mg^2+^, magnesium; min, minutes. **p<0.01.

Adipocytes were overexpressed with the Akt sensor (FoxO1-Clover) and stimulated with insulin. The translocation of FoxO1 from the nucleus to the cytosol was diminished in the Mg^2+^-deficient adipocytes compared to adipocytes incubated with a physiological Mg^2+^ concentration (P< 0.01) (**Figure 5**).

### Magnesium increases the insulin-stimulated glycolytic respiration rate

**Figure 6:**
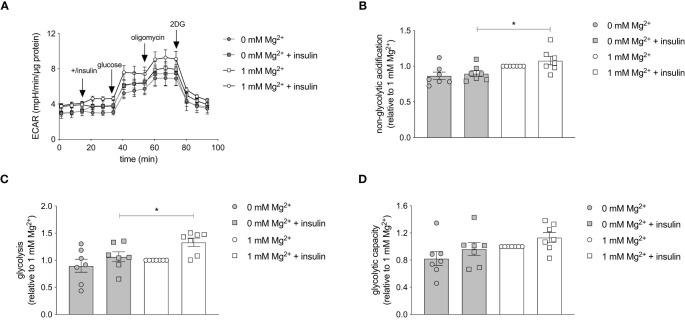
Effect of Mg^2+^ on insulin-stimulated glycolytic respiration rate in 3T3-L1 adipocytes. Glycolytic Stress Test in insulin-stimulated 3T3-L1 adipocytes incubated with 0 or 1 mmol/L Mg^2+^. **(A)** Representative raw Glycolytic Stress Test curve in 3T3-L1 adipocytes. Quantification of the **(B)** non-glycolytic acidification, **(C)** glycolysis and **(D)** glycolytic capacity in 3T3-L1 adipocytes (n=7). All data adjusted for protein. Legend: circle is without insulin, square is with insulin, grey color is 0 mmol/L Mg^2+^ and white color is 1 mmol/L Mg^2+^. Data are shown as mean ± S.E.M. Statistically analyzed with Two-Way ANOVA. *p<0.05. 2DG, 2-deoxyglucose; ECAR, extracellular acidification rate; Mg^2+^, magnesium; min, minutes.

The effect of Mg^2+^ on the intracellular energy metabolism was assessed using the Seahorse XF Glycolytic Stress Test. The glycolytic stress test curve demonstrated that the glycolysis is increased in insulin-stimulated adipocytes with a physiological level of Mg^2+^ compared to Mg^2+^-deficient adipocytes (**Figure 6A**). Oxygen consumption rate (OCR) was measured simultaneously and Mg^2+^ nor insulin-stimulation did not alter OCR rate (data not shown). The basal non-glycolytic acidification was higher in Mg^2+^-deficient adipocytes compared to adipocytes incubated with physiological Mg^2+^ concentration (P< 0.05) (**Figure 6B**), which suggests that increased glycolysis may have compensated for basal energy homeostasis. The glycolysis was enhanced in insulin-stimulated adipocytes compared to insulin-stimulated Mg^2+^-deficient adipocytes (P< 0.05) (**Figure 6C**). The glycolytic capacity (**Figure 6D**) remained the same in adipocytes incubated with and without Mg^2+^, with and without insulin stimulation.

## Discussion

The study demonstrated that Mg^2+^ plays an important role in insulin-dependent glucose uptake by increasing Akt activation and GLUT4 translocation to the plasma membrane. This may provide a mechanism by which a low Mg^2+^ may result in insulin resistance and explain the inverse association between, plasma Mg^2+^ concentration and insulin resistance in people with T2D.

Our results demonstrate that Mg^2+^ acts downstream of the IR and PI3K by activating Akt and/or GLUT4. Previous studies that investigated the effect of HIV1-protease inhibitors on insulin resistance are in agreement with our findings and demonstrated only signaling effects that are downstream of PI3K, in 3T3-L1 adipocytes and skeletal muscle (34–36). In non-insulin-stimulated macrophages and osteoblasts, Mg^2+^ activated Akt for anti-inflammatory properties and growth signaling (37, 38). All together, these findings suggest that insulin-dependent glucose uptake can be improved without affecting IR phosphorylation but can be driven by Akt activation and GLUT4 translocation. The functionality of Akt and GLUT4 have shown to be essential for preventing insulin resistance (39–41).

Animal studies that assessed the role of Mg^2+^ on PI3K/Akt signaling are limited and show inconsistent results. For instance, rats that are fed with a low Mg^2+^ diet showed diminished IR phosphorylation in skeletal muscle after only 4 days (42). While Reis et al. showed no differences on IR phosphorylation in skeletal muscle after a longer period of 11 weeks (43). Supplementation of Mg^2+^ in T2D rats did increase IR and GLUT4 expression levels in skeletal muscle (26, 27). However, these studies did not examine the intermediate PI3K/Akt signal transduction thoroughly in the skeletal muscle, thereby failed to show on which key proteins Mg^2+^ exerts in the skeletal muscle. We suggest that Mg^2+^ has a selective regulation of Akt-dependent processes that is cell-specific and differ in adipocytes compared to skeletal muscle (5).

The exact metabolic exertion of Mg^2+^ remains to be elucidated, but it is suggested that Mg^2+^ and ATP act together in the IR-PI3K-Akt pathway as a kinase substrate (44). Mg-ATP serves as substrate in the phosphoryl transfer reaction in the phosphorylation of proteins (45, 46). Additionally, Mg^2+^ may induce an electrostatic interaction, which enhances the binding affinity and stability of ATP to protein kinases (47). It has been established that Rab GTPases also have Mg^2+^-binding motifs (48), which may stimulate AS160 phosphorylation and GLUT4 translation.

The impaired glucose uptake caused by low intracellular Mg^2+^ affects the metabolism of the adipocyte. It has been well established that Mg-ATP acts as a co-factor for many key enzymes involved in glycolysis (49–51). In our study, glycolysis was decreased in Mg^2+^-deficient adipocytes compared to adipocytes incubated with a physiological Mg^2+^ concentration. In adipocytes, insulin stimulates the insulin-dependent glucose uptake and hexokinase and 6-phosphofructokinase activity, which both enhance glycolysis (52). The enhanced glycolysis could be related to increased activity of glycolytic enzymes (49), or can be a consequence of the increased insulin-dependent glucose uptake. We therefore suggest that the increased glycolysis, which may be a consequence of insulin-dependent glucose uptake, requires Mg^2+^ as a co-factor.

To date, Mg^2+^ studies have focused primarily on studying the insulin-dependent glucose uptake in skeletal muscles (26, 27, 42, 43). In contrast, we used mature 3T3-L1 adipocytes. The severity of insulin resistance in adipose tissue may even proceed skeletal muscle (53, 54), emphasizing the importance of identifying insulin sensitive mechanisms in adipose tissue. To our knowledge, there is one previous study demonstrating that Mg^2+^ deficiency reduced insulin-dependent glucose metabolism in isolated rat adipocytes, but this study did not assess the IR-PI3K-Akt pathway (55).

Our results suggest that Mg^2+^ deficiency in adipocytes exaggerates insulin resistance in people with T2D. Screening for hypomagnesemia and providing Mg^2+^ supplementation may improve insulin sensitivity, which prevents the development of T2D (23, 56).

A strength of our study is that we examined a large part of the IR-PI3K-Akt pathway. On the other hand, a limitation of our approach is that only two concentrations of Mg^2+^ was used in our study, namely a cell physiological concentration (1 mmol/L Mg^2+^) and Mg^2+^-deficient (0 mmol/L Mg^2+^). Additionally, the intracellular Mg^2+^ concentration and other electrolytes were not measured, limiting conclusions on the direct metabolic effect of Mg^2+^. It should be noted that a short-term (24 hours) incubation with 0 mmol/L Mg^2+^ was selected to cause a significant reduction of the intracellular Mg^2+^ concentration. Experiments in HEK293 cells have demonstrated that intracellular Mg^2+^ levels are generally decreased within a few hours (57). Furthermore, a reduction of intracellular Mg^2+^ is observed when incubating myocytes (58), Madin-Darby Canine Kidney (MDKC) (59), mouse distal convoluted tubule (60), or breast cancer MDA-MB-231 cells (57) for 16-24 hours with 0 mmol/L Mg^2+^ (61). As such, our approach could be representative for the chronic low cytosolic free intracellular Mg^2+^ levels in people with T2D and hypomagnesemia.

In summary, Mg^2+^ acts on Akt and GLUT4 to improve insulin signaling and glucose uptake in adipocytes; thereby controlling extracellular glucose levels. The increased availability of glucose is translated into an increased glycolysis in adipocytes. The defects in Akt and GLUT4 signaling by Mg^2+^ deficiency could be the fundamental reason for the negative association of hypomagnesemia and insulin resistance in people with T2D.

## Data availability statement

The original contributions presented in the study are included in the article/supplementary material. Further inquiries can be directed to the corresponding author.

## Ethics statement

The studies involving human participants were reviewed and approved by the Ethics committee of the Radboud University Medical Center. The patients/participants provided their written informed consent to participate in this study.

## Author contributions

LO and SK performed together the radioactive glucose uptake experiments. LO performed all the other experiments described in the manuscript. SK and CM did preliminary analysis that were essential for the set-up of this study. LO and JB wrote the manuscript. JH, CT, and JB supervised the study. All authors reviewed and approved the final version of the manuscript. JB is the guarantor of this work and had full access to all the data in the study and takes responsibility for the integrity of the data.

## Funding

This research was funded by the Dutch Diabetes Research Foundation (2017–81–014).

## Conflict of interest

The authors declare that the research was conducted in the absence of any commercial or financial relationships that could be construed as a potential conflict of interest.

## Publisher’s note

All claims expressed in this article are solely those of the authors and do not necessarily represent those of their affiliated organizations, or those of the publisher, the editors and the reviewers. Any product that may be evaluated in this article, or claim that may be made by its manufacturer, is not guaranteed or endorsed by the publisher.
